# Optimizing Distribution of Pandemic Influenza Antiviral Drugs

**DOI:** 10.3201/eid2102.141024

**Published:** 2015-02

**Authors:** Bismark Singh, Hsin-Chan Huang, David P. Morton, Gregory P. Johnson, Alexander Gutfraind, Alison P. Galvani, Bruce Clements, Lauren A. Meyers

**Affiliations:** The University of Texas at Austin, Austin, Texas, USA (B. Singh, H.-C. Huang, G.P. Johnson, L.A. Meyers);; Northwestern University, Evanston, Illinois, USA (D.P. Morton);; University of Illinois at Chicago Illinois, USA (A. Gutfraind);; Yale University, New Haven, Connecticut, USA (A.P. Galvani);; Texas Department of State Health Services, Austin (B. Clements)

**Keywords:** antiviral drugs, influenza, influenza virus, viruses, 2009 pandemic, influenza A(H1N1)pdm09 virus, pandemic influenza, optimization, control, pharmacies, pharmacy-based drug distribution, underinsured populations, ZIP codes, Texas

## Abstract

We provide a data-driven method for optimizing pharmacy-based distribution of antiviral drugs during an influenza pandemic in terms of overall access for a target population and apply it to the state of Texas, USA. We found that during the 2009 influenza pandemic, the Texas Department of State Health Services achieved an estimated statewide access of 88% (proportion of population willing to travel to the nearest dispensing point). However, access reached only 34.5% of US postal code (ZIP code) areas containing <1,000 underinsured persons. Optimized distribution networks increased expected access to 91% overall and 60% in hard-to-reach regions, and 2 or 3 major pharmacy chains achieved near maximal coverage in well-populated areas. Independent pharmacies were essential for reaching ZIP code areas containing <1,000 underinsured persons. This model was developed during a collaboration between academic researchers and public health officials and is available as a decision support tool for Texas Department of State Health Services at a Web-based interface.

## Abstract 2

Effective distribution of these drugs will reduce illness and death in underinsured populations.

Influenza pandemics occur when novel strains of the influenza virus emerge in human populations and spread worldwide ( *1* ). There were 3 influenza pandemics in the 20th century (1918, 1957, and 1968), and 1 has occurred so far in the 21st century (2009). The 1918 Spanish flu pandemic was far more severe than the others, causing an estimated 50 million deaths globally ( *2* ). In contrast, the 2009 pandemic had an estimated death toll of 284,000 ( *3* ). Experts conjecture that the risk for new pandemics will increase in the coming decades ( *4* ), and several emerging threats are already under surveillance. A highly pathogenic avian influenza A (H5N1) virus has occasionally been infecting humans in Asia, Africa, and Europe since 1997 ( *5* ); the first human case in North America was reported in January 2014 ( *6* ). Since March 2013, China has been trying to contain an ongoing outbreak of a highly pathogenic avian influenza (H7N9) virus ( *7* , *8* ).

The primary control measures for pandemic influenza are antiviral medications and vaccines ( *9* ), as well as nonpharmaceutical interventions, such as social distancing measures, school closures, and hygienic precautions ( *10* ). Although the efficacy of influenza vaccines depends on factors such as patient age and virus type/subtype ( *11* ), these vaccines are arguably the best intervention strategy ( *1* ). However, because development and deployment of effective vaccines for a new influenza virus may take several months ( *12* ), antiviral drugs and nonpharmaceutical interventions are particularly critical for early pandemic control.

Antiviral drugs are believed to reduce disease severity and duration of infectiousness in individual patients, if taken sufficiently early ( *9* ), and to protect contacts of infected persons, if taken prophylactically ( *13* *–* *15* ). Some studies have suggested that aggressive treatment policies can effectively mitigate local transmission ( *16* , *17* ). In preparation for future influenza pandemics, the US Department of Health and Human Services therefore maintains a large Strategic National Stockpile (SNS) of antiviral drugs ( *18* ), and most states include SNS antiviral drugs as a major component of their pandemic response plans ( *19* *–* *22* ).

After detection of the new influenza A(H1N1)pdm09 virus in April 2009, the US government declared a public health emergency, and the World Health Organization declared a global influenza pandemic. Vaccines became widely available after 6 months of sustained transmission ( *23* ). In the early weeks of the pandemic, the US Department of Health and Human Services distributed 11 million courses of federally held SNS antiviral drugs to states ( *24* ) and issued a series of guidelines for implementing antiviral drug and nonpharmaceutical interventions. During the pandemic, many states sought to work in cooperation with retail pharmacies and independent drug stores to assist in dispensing their shares of the SNS and state caches ( *19* *–* *22* ). The Texas Department of State Health Services (DSHS) enlisted the help of several major pharmacy chains and independent retail pharmacies to dispense >200,000 antiviral drug courses from the SNS and state cache ( *25* ). A report analyzing the pandemic response indicated that additional planning is required to ensure that persons residing in counties in Texas lacking pharmacies can obtain antiviral drugs when needed ( *26* ).

We propose a method for optimizing the location of dispensing points for antiviral drugs within a state. Several state pandemic response plans include the following goals: using commercial pharmacies as antiviral drug-dispensing partners to limit strain on hospitals; reaching a population of broad demographics, including underinsured populations; and improving convenience. These states include Virginia ( *19* ), Louisiana ( *20* ), Florida ( *21* ), Tennessee ( *22* ), and Texas ( *25* ). These plans further specify that the choice of participating chains will depend on their location and local demographics, and they include strategies for reaching the underinsured population at minimal or no cost.

Working with Texas DSHS, we developed a data-driven facility-location model for designing commercial pharmacy antiviral drug distribution networks that maximizes access to underinsured populations, when access is based on a willingness-to-travel model estimated from National Household Travel Survey data ( *27* ). We describe the model that is now available to the Texas DSHS as a Web-based decision-support tool for future pandemics ( *28* ), and we use it to evaluate and optimize the commercial pharmacy distribution network established in Texas during the 2009 influenza pandemic.

## Methods

### Data

Texas has 1,939 US postal code (ZIP code) areas in 254 counties; 1,023 of these ZIP code areas contain ≥1 pharmacy ( Table 1 ). We obtained the addresses of all community and clinic pharmacies with active licenses listed by the Texas Pharmacy Board ( *29* ). The largest chains (present in the most ZIP code areas) in Texas are Brookshire, Costco, CVS, HEB, Kmart, Kroger, Randalls, Sam’s Club, Target, Tom Thumb, United, Walgreens, and Walmart. Other pharmacies, independent or small chain, are listed as independents. The Texas DSHS provided the list of pharmacies selected to dispense antiviral drugs to underinsured populations during the 2009 influenza pandemic; these pharmacies were in 723 ZIP code areas. To approximate the size of the uninsured and underinsured population in each ZIP code area (direct statistics were not available), we used the number of persons in households with an annual income <$20,000 ( http://www.bio.utexas.edu/research/meyers/_docs/publications/SinghEID14Supplement.pdf ).

**Table 1:** Number of ZIP code areas containing ≥1 pharmacy of each chain and geographic overlap with Walgreens, which is present in the most ZIP code areas, Texas, USA*

Pharmacy name	No. ZIP code areas	Overlap with Walgreens, %
Walgreens	490	100.0
CVS/Pharmacy	422	75.1
Walmart	372	68.8
HEB	189	77.8
Kroger	167	86.8
Target	127	86.6
Brookshire	118	28.0
Sam’s Club	74	89.2
Tom Thumb	51	74.5
Randalls	42	90.5
United	40	50.0
Costco	20	80.0
Kmart	16	81.3
Independents	893	48.0

*ZIP code areas, US postal code areas.

Our optimization model uses a geographic resolution of ZIP code areas based on ZIP code tabulation areas (ZCTAs) ( *30* ). ZCTAs differ slightly from US Postal Service ZIP code areas and may include ≥1 US Postal Service ZIP code area. We mapped each pharmacy and residential ZIP code area to its corresponding ZCTA ( *31* ), and, for simplicity, we refer to these as ZIP code areas.

### Willingness-to-Travel Model

**Figure 1:**
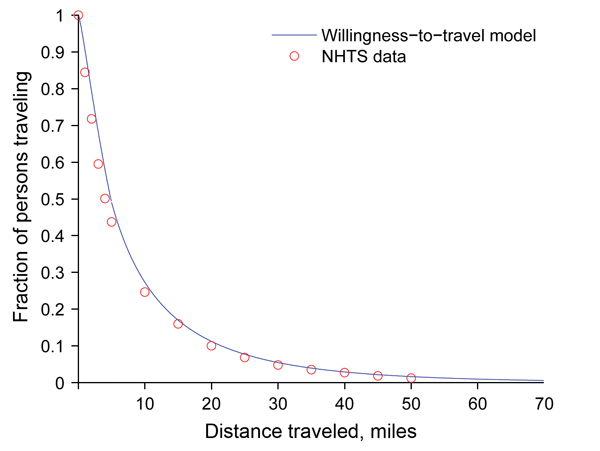
Willingness-to-travel curve for receiving antiviral drugs during the 2009 influenza pandemic given by equation (2) (in Methods section) fit to National Household Travel Survey (NHTS) data on privately operated vehicle travel for the entire US underinsured population.

We used National Household Travel Survey (NHTS) data for 2009 ( *27* ) to estimate the distances persons are willing to travel in Texas to obtain antiviral drugs sufficient for a course of treatment during an influenza pandemic (model described below). We created a willingness-to-travel model, which follows an exponentially decaying distribution, by fitting the model to national-scale NHTS data for privately operated vehicle travel ( *27* ) ( Figure 1 ). This included ≈330,000 person trips (83% of all person trips in the database), totaling 3.3 million miles, including ≈30,000 person trips originating in Texas. We made the simplifying assumption that health care–seeking behavior in Texas during an influenza pandemic will resemble national willingness to travel by privately operated vehicle for work, school, family, and social reasons. Although there are probably major differences in these estimates, we believe that this model conservatively underestimates actual accessibility of pharmacies during a pandemic.

in which the *P* ( *d* ) term is the fraction of the target population willing to travel at least *d* miles. As the required travel distance increases, the fraction of the population willing to travel distance *d* decreases. We used a piecewise model that allows for different coefficients below and above a distance threshold of 5 miles to enable urban and rural populations to exhibit different willingness-to-travel patterns ( http://www.bio.utexas.edu/research/meyers/_docs/publications/SinghEID14Supplement.pdf ).

To estimate travel patterns for the underinsured population, we considered NHTS data for households with incomes <$20,000 ( http://www.bio.utexas.edu/research/meyers/_docs/publications/SinghEID14Supplement.pdf ) and found that the travel patterns for this group are given by

The estimated willingness-to-travel for the underinsured population is slightly greater (<1%) than that for the entire population. The adjusted *R^2^* values for each model exceed 0.99.

### Optimization Model

The optimization model we used identifies ZIP code areas for pharmacy-based distribution of SNS and state-cache antiviral drugs to maximize access in the target population (either underinsured or entire population). It is a facility-location type model ( *32* *,* *33* ) with an objective function defined in terms of the expected number of persons willing to obtain antiviral drugs from the nearest dispensing point. We estimated this quantity by using our willingness-to-travel model for the distance between the home ZIP code centroid and pharmacy ZIP code centroid. For the distance to a pharmacy within the home ZIP code area, we used a correction factor based on the size of the ZIP code area ( http://www.bio.utexas.edu/research/meyers/_docs/publications/SinghEID14Supplement.pdf ).

The optimization model takes as input the total number of ZIP code areas to be included in the distribution network ( *b* ). The model does not account for the number of available antiviral drug doses, the number to be shipped to each pharmacy, or the capacity of individual pharmacies. Additional details on methods are available at http://www.bio.utexas.edu/research/meyers/_docs/publications/SinghEID14Supplement.pdf . 

**Figure 2:**
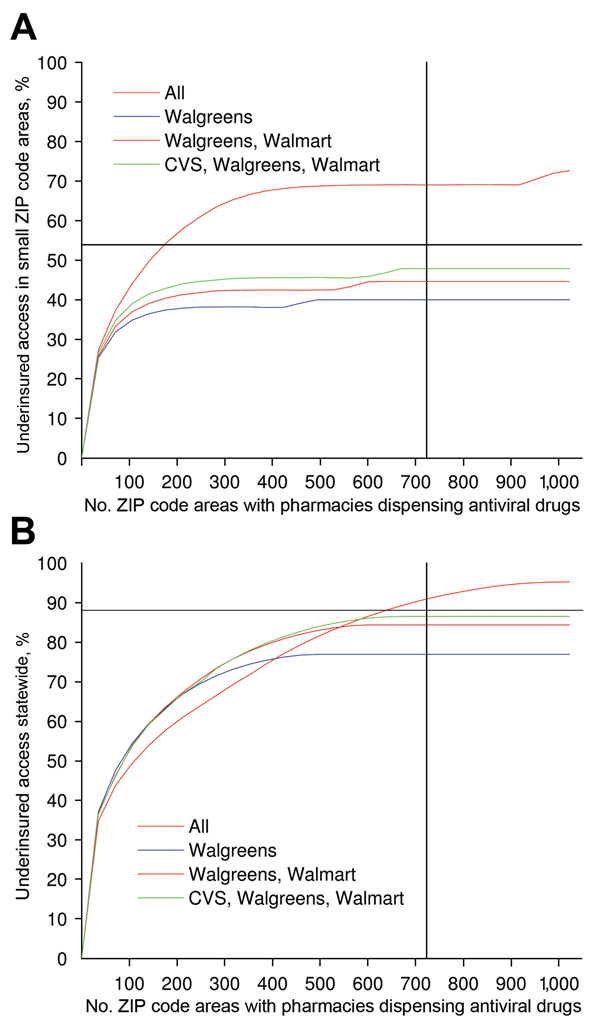
Antiviral drug access in underinsured populations achieved by the Texas antiviral drug distribution network during the 2009 influenza A pandemic and by optimized antiviral drug distribution networks, for A) small ZIP code (US postal code) areas (i.e., ZIP code areas with <1,000 underinsured persons) and B) statewide. Access is the expected fraction of the underinsured population willing to travel to the nearest dispensing pharmacy to obtain antiviral drugs. The black vertical and horizontal lines indicate the number of ZIP code areas that participated in the Texas 2009 distribution network and the estimated access achieved, respectively. For each network size (number of dispensing ZIP code areas), a hybrid optimization was performed to maximize coverage in small ZIP code areas and overall (see Methods for details). Color indicates which combination of 13 major pharmacy chains plus independents were considered in the optimization. For a distribution network of size 723 (comparable to the Texas 2009 H1N1 antiviral drug distribution), the best performing single-chain (Walgreens), 2-chain combination (Walgreens and Walmart), and 3-chain combination (Walgreens, Walmart, and CVS) provided near optimal coverage statewide, but critically underserved the smallest ZIP code areas.

The Web-based decision-support tool based on this model provides solutions for a range of values of *b* ( Figure 2 ) and displays the trade-off between the expected access for the target population and the number of dispensing points. This tool also enables the user to select specific solutions for further analysis.

We considered 3 types of objective functions, all of which focus exclusively on the underinsured population in Texas: maximizing statewide access, maximizing access in small ZIP code areas (i.e., ZIP code areas with <1,000 underinsured persons), and a hybrid that combines the first 2 objectives. For our hybrid optimization model, we first specified a percentage of all dispensing points to focus on small ZIP code areas ( *P* ). Second, we optimized *P* of all dispensing points solely for access in small ZIP code areas and recorded the access achieved in small ZIP code areas ( *A_s_* ). Third, we started over and optimized all dispensing points by using the statewide objective function with the added constraint that the solution must achieve a minimum of 0.95 *A_s_* access in small ZIP code areas. This method simultaneously achieves near maximal coverage statewide and in small ZIP code areas.

## Results

During the 2009 influenza pandemic, the Texas DSHS recruited 1,393 pharmacies from 6 major chains and 71 independent pharmacies to dispense antiviral drugs from the SNS and state cache to underinsured populations. These pharmacies were located in 723 of the 1,023 ZIP code areas in Texas that had ≥1 pharmacy. We estimated that this network provided antiviral drug access for 88% of the state’s underinsured population ( Figure 2 ). In comparison, we also estimated that optimization over all possible pharmacy chains produced a network expected to achieve comparable access by using only 526 ZIP code areas, increased access to 92.5% with 723 ZIP code areas, and reached a maximum access of 95.2% with all 1,023 ZIP code areas.

However, optimizing for statewide access can lead to critical gaps in coverage. We categorized all Texas ZIP code areas on the basis of underinsured population sizes into small (<1,000 persons), medium (1,001–7,000 persons), and large (>7,000 persons). These areas contained 7%, 51%, and 42% of the statewide underinsured population, respectively. The actual Texas 2009 distribution network and the corresponding optimized network (with 723 ZIP code areas) were estimated to achieve only 34.5% and 38.3% access in small ZIP code areas, respectively, but reached 88.0% and 92.5% access overall. By definition, the small ZIP code areas do not carry much weight in a statewide optimization model. They also tend to be more remote than larger ZIP code areas, and thus have lower access to selected pharmacies.

To address this gap, we modified the objective function to maximize access specifically in small ZIP code areas. Although these modifications improved coverage in these hard-to-reach populations, the solutions were suboptimal overall. Thus, we developed a hybrid optimization procedure that sequentially ensures high access statewide and in small ZIP code areas. With 723 dispensing points, the hybrid method with *P* = 75% of dispensing points allocated to small ZIP code areas produced networks that are expected to achieve 60.5% access in small ZIP code areas and 90.5% overall. For comparison, the highest possible access (when all pharmacies in the state dispense antiviral drugs) was estimated to reach 63.8% and 95.2% in the 2 populations, respectively ( Table 2 ). For populations living in ZIP code areas without pharmacies dispensing antiviral drugs, optimization reduced the average travel distance to the nearest dispensing pharmacy from 4.5 miles to 3.8 miles.

**Table 2:** Expected access for antiviral drugs during the 2009 influenza pandemic provided by 3 drug distribution networks, Texas, USA*

Characteristic	Texas 2009 network	Optimized network†	All pharmacies network
Small ZIP code area access, %	34.5	60.5	63.8
Statewide access, %	88.0	90.8	95.2
No. ZIP code area dispensing points	723	723	1,023
Population living within dispensing ZIP code areas, %	76.5	79.3	91.8
Average miles traveled outside ZIP code area (SD)‡	4.5 (3.8)	3.8 (3.1)	5.7 (4.0)
Median miles traveled outside ZIP code area§	3.0	2.6	4.2

*ZIP code area, US postal code area. A small ZIP code area is an area with <1,000 underinsured persons. †Initially, we optimized 75% of dispensing points (542) to maximize access solely in small ZIP code areas and recorded the access achieved. We then optimized all 723 dispensing points to maximize statewide access and constrained the solution to achieve ≥95% of the small ZIP code area access achieved in the initial optimization. ‡Population-weighted average travel distance to nearest dispensing pharmacy considering only ZIP code areas without their own dispensing pharmacies. When all pharmacies dispense antiviral drugs (fourth column), the longer average distance is an artifact of few persons living in ZIP code areas without pharmacies. §Population-weighted median travel distance to nearest dispensing pharmacy, considering only ZIP code areas without their own dispensing pharmacies. When all pharmacies dispense antiviral drugs (fourth column), the longer median distance is an artifact of few persons living in ZIP code areas without pharmacies.

**Figure 3:**
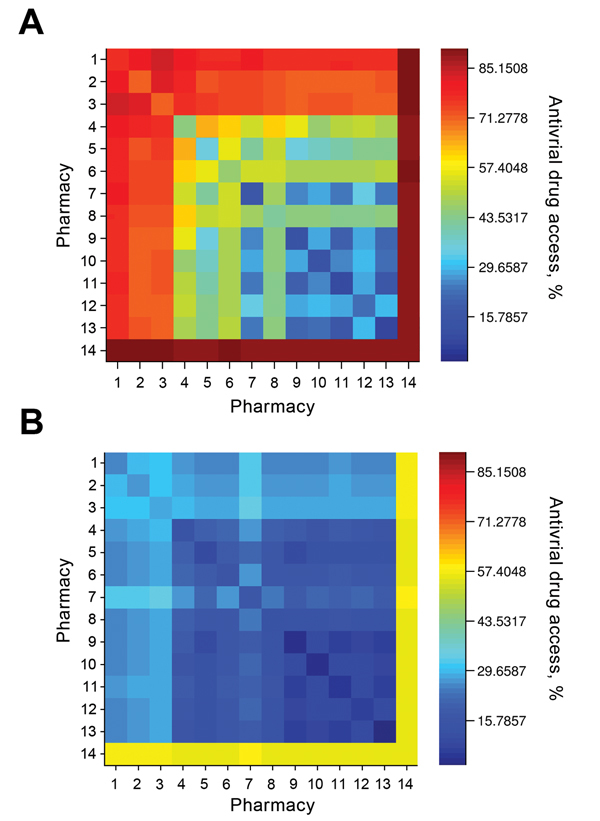
Antiviral drug access in underinsured populations for single-chain and 2-chain pharmacy distribution networks during the 2009 influenza pandemic, Texas, USA. Each network contains a maximum of 723 distribution points, and was designed by using a hybrid optimization that maximizes coverage in small ZIP code (US postal code) areas and overall (see text for details). Color indicates the expected percentage of the underinsured population willing to travel to dispensing pharmacies to obtain antiviral drugs A) statewide and B) in small ZIP code areas. Numbers along the baselines and the y-axes indicate single-chain networks. 1, Walgreens; 2, CVS; 3, Walmart; 4, HEB; 5, Kroger; 6, Target; 7, Brookshire; 8, Sam’s Club; 9, Tom Thumb; 10, Randalls; 11, United; 12, Costco; 13, Kmart; 14, Independents (independent pharmacies).

A state might opt to limit the number of chains in the distribution network to simplify logistics. The pharmacy chains eligible for participation in Texas include Brookshire, Costco, CVS, HEB, Kmart, Kroger, Randalls, Sam’s Club, Target, Tom Thumb, United, Walgreens, Walmart, and independent retail pharmacies (henceforth independents). During the 2009 influenza pandemic, the Texas DSHS distributed antiviral drugs from the SNS and state cache through 6 major chains (Brookshire, HEB, Kroger, United, Walgreens, and Walmart), and independents. When we restricted the optimization to a few major chains, the resulting networks still achieved broad statewide coverage ( Figures 2 , 3 ). For example, Walgreens alone was expected to achieve ≈75% coverage if it dispenses in all of its 490 ZIP code areas; CVS and Walmart followed close behind (located in 422 and 372 ZIP code areas, respectively). These 3 chains have the greatest presence in the state ( Table 1 ), but have highly overlapping geographic areas. Broad accessibility can also be achieved through a combination of smaller chains with geographic complementarity, for example, HEB and Kroger (in 189 and 167 ZIP code areas, respectively). Walgreens and Walmart overlap over half of their ZIP code areas (256), whereas HEB and Kroger overlap in only 30 ZIP code areas. However, the number of ZIP code areas alone is not predictive of access. For example, Brookshire has almost as many stores as Target (in 118 and 127 ZIP code areas, respectively), yet provides considerably less statewide access alone and in combination with other stores.

**Figure 4:**
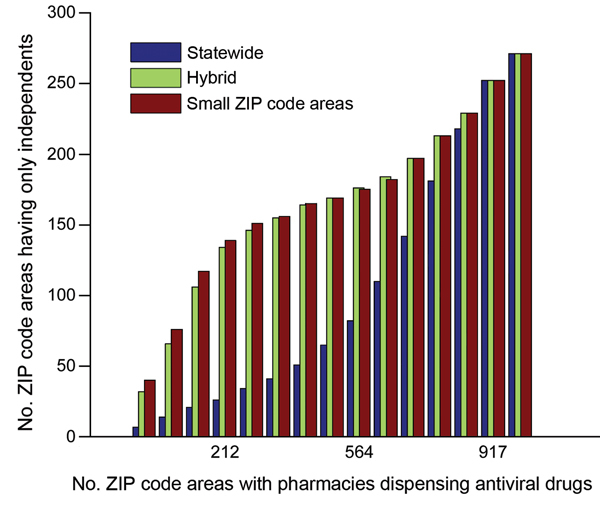
Number of sites in the antiviral drug distribution network during the 2009 influenza pandemic that contained only independent pharmacies (independents; i.e., no major chains) when optimizing for the underinsured population in small ZIP code (US postal code) areas (i.e., ZIP code areas with <1,000 underinsured persons), statewide, or both (hybrid), Texas, USA.

However, the major chains did not reach the underinsured populations in the small ZIP code areas, even under the hybrid optimization that explicitly targets these hard-to-reach populations ( Figure 2 , panel A). Independent pharmacies are essential to bridging this gap in coverage. The maximum access achieved by a 2-chain combination in small ZIP code areas is only 33% (Brookshire and Walmart) ( Figure 3 ). Under the hybrid objective, optimized networks with <500 dispensing points yield solutions for all major pharmacy chains plus independents that provided slightly lower statewide accessibility than the corresponding solutions for major chains ( Figure 2 , panel B), in exchange for higher coverage in small ZIP code areas ( Figure 2 , panel A). Of the 1,023 ZIP code areas with ≥1 pharmacy, 271 have only independent pharmacies. Of these pharmacies, 167 are in small ZIP code areas, and were typically selected when optimizing for access in small ZIP code areas, but not when optimizing for access statewide ( Figure 4 ).

## Discussion

**Figure 5:**
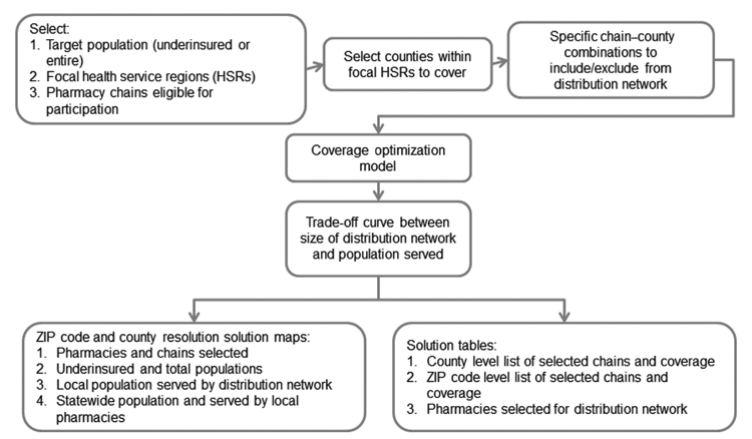
Flowchart of the antiviral drug distribution decision support tool during the 2009 influenza pandemic ( *28* ), Texas, USA.

**Figure 6:**
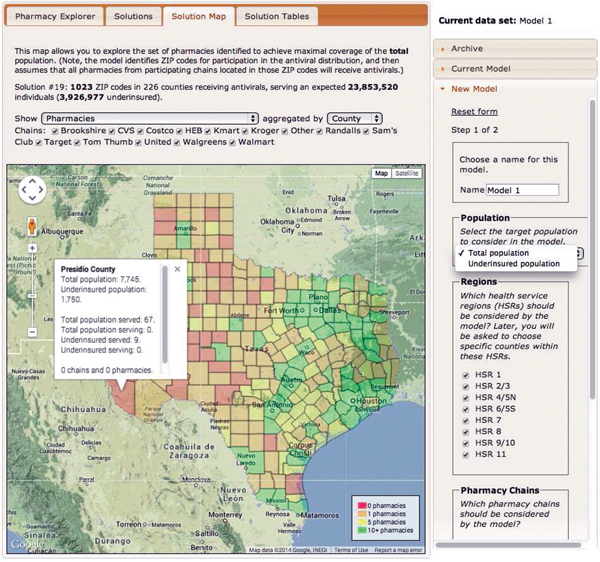
Screenshot of the antiviral drug distribution decision support tool used during the 2009 influenza pandemic ( *28* ), Texas, USA.

Many states plan to enlist commercial pharmacies in the dispensing of SNS antiviral drugs during future influenza pandemics ( *20* *–* *22* *,* *34* ). We have developed and demonstrated a simple, extensible, facility-location model for designing pharmacy-based antiviral drug distribution networks that effectively reach target populations. This model has been parameterized for the state of Texas and incorporated into a Web-based decision-support tool ( *28* ) for the Texas DSHS ( Figures 5 , 6 ). The user can opt to target the underinsured or total population statewide or within any specified counties and to exclude or include particular pharmacies and pharmacy chains. On the basis of user input, the tool solves a family of optimization models, spanning the full range of possible network sizes, and presents the structure and performance of the optimized networks by using interactive graphs and maps. The tool is designed for use by Texas DSHS staff, who have Web-based access to it. Although this implementation is specific for Texas, the general model structure is readily adaptable to other jurisdictions. Adaptation requires specification of geographic units (e.g., ZIP code areas or counties), distances between each pair of units, estimated target population sizes within each unit, and number of pharmacies from each eligible chain located within the unit. The model can be easily extended to other states by using data available through the US Census Bureau and state pharmacy associations ( *29* , *35* ).

The optimization model is driven by a willingness-to-travel model, which was estimated from NHTS data. The nature and resolution of available data led to several simplifying assumptions. After fitting several decaying functions to the data, we chose a simple model that considers only the distance between one’s home ZIP code area and the nearest pharmacy ZIP code area, rather than, for example, a more complex gravity model that incorporates the attractiveness of a store ( *36* , *37* ). The national data did not specify health-related travel patterns. Texas NHTS data had coarser mileage bins, and represented only 1% of the nation-wide person-trips. Thus, we used national-scale data on all available travel categories (to earn a living, family/personal business, school/church, social/recreational, and miscellaneous), and included only travel by privately operated vehicles (because of the sparsity of data on public transportation and bicycles). We also approximated home ZIP code area distances to pharmacy ZIP code area distances by using great circle distances between the ZIP code centroids, rather than road travel distances between their street addresses, which can underestimate the distance an individual must travel. Finally, we approximated the underinsured and small ZIP code area populations by using methods described in the Data section.

During future pandemics, timely and effective deployment of antiviral drugs from the SNS and state cache might be essential for reducing early illness and death ( *19* ). Data-driven models, such as those in this optimization tool can be instrumental in the planning process, enabling public health agencies to identify and recruit networks of broad-reaching chains and remote independent pharmacies that can achieve equitable and effective distributions to target groups, such as underinsured, high-risk, or age-specific populations. This tool will also facilitate rapid, adaptive decision-making during pandemics, if, for example, a region requires additional supplies, particular pharmacies are unwilling or unable to provide assistance, or the target population changes.

As with many optimization studies, the general insights gleaned from the design and preliminary applications of this decision-support tool might be as valuable as the tool itself. The Texas DSHS has gained actionable perspectives on the geographic coverage and redundancies of major pharmacy chains, the unique reach of independent pharmacies, and as discussed further ( http://www.bio.utexas.edu/research/meyers/_docs/publications/SinghEID14Supplement.pdf ), the convenient overlap between optimal distribution networks for the underinsured population and total population. Given the need for and difficulties associated with enlisting independent pharmacies in sparsely populated areas, state agencies should engage them well before the next pandemic, perhaps in partnership with local health departments.
